# Role of m6A modification in female infertility and reproductive system diseases

**DOI:** 10.7150/ijbs.69771

**Published:** 2022-05-16

**Authors:** Jinyu Chen, Yiwei Fang, Ying Xu, Haotong Sun

**Affiliations:** Institute of Reproductive Health, Tongji Medical College, Huazhong University of Science and Technology, Wuhan, Hubei, 430030, P.R. China.

**Keywords:** RNA modification, N6-methyladenosine, Female reproductive diseases, Infertility, Reproductive system neoplasms

## Abstract

Gamete abnormalities and reproductive system tumors have become a dominant cause of infertility, troubling people globally. In recent years, increasing evidence emerged and found that N6-methyladenosine (m6A) played a leading role in reproduction. The biological effects of m6A modification are dynamically and reversibly regulated by methyltransferases (writers), WTAP, METTL3, METTL14 and KIAA1429, demethylases (erasers), FTO and ALKBH5, and m6A binding proteins (readers), including YTH domain. In this review, we highlight the change of m6A modification in abnormal oogenesis, female reproductive system diseases including reproductive system tumors, adenomyosis, endometriosis, premature ovarian failure and polycystic ovary syndrome. Moreover, we review some of the mechanisms and the specific modified genes that have been identified. Especially, with the underlying mechanisms being uncovered, m6A and its protein machineries are expected to be the markers and targets for the diagnosis and treatment of female reproductive dysfunction.

## Introduction

Recent studies have revealed that epigenetic modification of diseases has emerged as an important regulator of a variety of physiological processes and disease progression, attracting accumulating attention in bioscience research. Epigenetic processes, including DNA methylation, histone modifications, chromatin rearrangement, and RNA modifications, play crucial roles in the regulation of many physiological and pathological processes, such as embryonic development 1, nervous system development 2, and tumorigenesi*s* 3. Among them, RNA modification comes into public view in recent years. There are numerous types of RNA modifications, of which more than 160 have been discovered up to now 4. Studies have widely reported certain types of RNA modifications in eukaryotic mRNA, including m6A, N1-methyladenosine, and 5methylcytosine. m6A is the most abundant internal modification of RNA in the majority of eukaryotes. Since the pioneering research in the 1970s 5, with the identification of more m6A-related enzymes, the important biological functions played by m6A modification have been gradually revealed around about half a century later. Besides, the rapid development of m6A detection technology pushes m6A research to a new height. Called m6A iCLIP (miCLIP), an individual-nucleotide resolution UV crosslinking and immunoprecipitation (iCLIP)-based method was born in 2015, which allows the transcriptome-wide mapping of individual m6A residues at single-nucleotide resolution 6. The advance in miCLIP2 results in high-complexity miCLIP2 libraries using less input material at less effort 7. Encouragingly, single-base extension and linkage qPCR amplification technology could greatly shorten the detection time of m6A level, and could uncover specific m6A modified genes and their targets, paving the way for the possibility of m6A as a diagnostic method. More recently, the technique of SELECT-m6A modified quantitative detection is gradually mature 8.This technological advance opens up the possibility of m6A being involved in the study and diagnosis of diseases.

**Figure 1:**
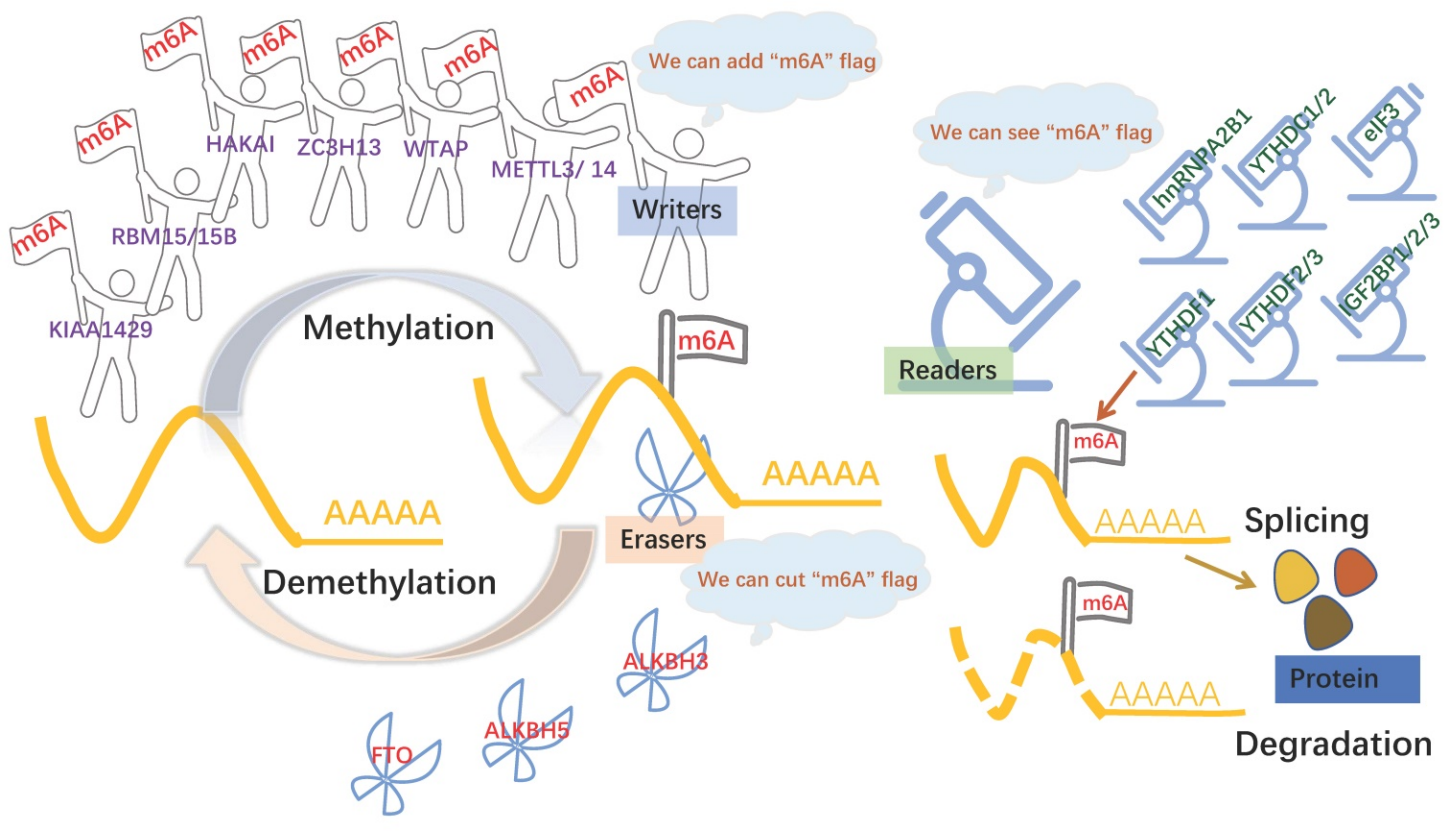
** m6A modification is regulated by 3 components.** m6A modification is added by “writers”, such as METTL3, METTL14, KIAA1429, WTAP. m6A could be reversibly removed by “erasers” (FTO and ALKBH5) or recognized by m6A binding proteins (“readers”, such as YTHDC1/2, YTHDF1/2/3 and IGF2BP1) to influence RNA splicing and degradation.

The formation of m6A is a dynamic and reversible process 9, m6A “writers” with methyltransferase activity are consisted of three individual proteins: methyltransferase-like (METTL) -3, METTL14, Wilms-tumor associating protein (WTAP), Vir-like m6A methyltransferase-associated (VIRMA; also known as KIAA1429) 10. The methyltransferase complex catalyzed m6A modification by METTL3 and METTL14 and a regulatory subunit WTAP. METTL3 was first shown to be m6A methylation transferase, whose expression can direct effect the total methylation level of m6A, which has effects on mRNA stability, leading to dysregulated cellular functions 11. METTL14 forms a stable complex with METTL3 and plays a key role in substrate recognition 12. WTAP regulates transcription and translation of niche factors by depositing the m6A marks directly on transcripts encoding the niche factors or indirectly on transcription 13. Obesity-associated protein (FTO) and alkB homolog 5 (ALKBH5), m6A demethylases, are able to mediate that methylation reversal through getting rid of the m6A modification 14. Another protein machineries functioned as m6A “readers,” including YTH domain family proteins (YTHDFs) and YTH domain-containing proteins 1-2 (YTHDCs), the insulin-like growth factor 2 mRNA binding proteins (IGF2BPs) 15, heterogeneous nuclear ribonucleoprotein A2B1 (HNRNPA2B1) 16, and eukaryotic initiation factor 3 (eIF3) 17*,* which can recognize m6A modification to modulate mRNA fate^18^. For example, YTHDF1 promotes the translation of m6A modified mRNA, while YTHDF2 lowers mRNA stability, induces mRNA degradation, and mediates mRNA subcellular localization and selective splicing. (**Figure 1**) The other types of m6A protein machineries have been introduced in detail in a large number of reviews 19, 20.

Studies have reviewed the functions and roles of m6A protein machineries in diverse diseases, such as acute myeloid leukemia, glioblastoma, lung cancer, liver cancer 21, nonalcoholic fatty liver disease 22, azoospermia 23, heart failure 24. m6A modification also plays an important role in eukaryotes 25 and cell proliferation and differentiation 26. Recently, studies have revealed the role of m6A modification and its protein machineries in oogenesis and female reproductive tumors and other female reproductive diseases. In oogenesis, the lack of YTHDF2 leads to the failure of m6A modified mRNA degradation, which affects the quality of oocytes 27. In reproductive tumors, METTL3 is upregulated in ovarian cancer (OC) 28.

The incidence and prevalence of infertility and female reproductive system tumors increase steadily worldwide and have become a prevalent worldwide problem in recent decades. Among them, abnormal oogenesis in infertility accounted for a large proportion. So far, there is no better treatment for infertility caused by abnormal gametes. For female reproductive system tumors, targeted drug therapy is one of the important treatments, but there may be adverse consequences, as well as drug resistance. Therefore, the exploration for better treatment is urgent. Other diseases that severely impair female health and may cause infertility are endometriosis, premature ovarian failure, polycystic ovarian syndrome (PCOS) and adenomyosis. Until now, the pathogenesis of endometriosis and PCOS is still not well understood, and there is no effective treatment. Therefore, greater insights into the mechanisms regulating spermatogenesis and male genital system tumors will help us found novel molecular targets to develop more effective treatment strategies for these diseases.

So far, few reviews have addressed m6A in relation to female reproductive health. Hence, we summarize and focus on the role of m6A modification and its protein machineries in oogenesis and female reproductive system diseases including tumors and PCOS and so on. Moreover, we also review some of the revealed mechanisms and specific genes modified by m6A, desiring to explore the possibility that some m6A target sites could be used to diagnose and treat reproductive disorders.

## Mechanisms of m6A Protein Machinery

As the most common and extensive base modification method at the RNA level, m6A methylation profoundly influences all aspects of mRNA-associated processes. m6A modification is affected by m6A protein machinery. So m6A protein machinery can influence mRNA-associated process, including alternative splicing, nuclear export, translation, and stability.

### m6A in mRNA splicing

In the term of alternative splicing, m6A modification regulates gene expression by interfering with this process. m6A methylation that directly influences splicing is usually located near exonic or intronic splice junctions, matching its function. METTL3 dependent m6A modification has little effect on alternative splicing. Instead, m6A-regulated splicing is rapid and dynamic in changing environments and under pathological conditions. It only occurs under specific circumstances, rather than functioning as a wide-ranging regulatory event that persists under normal physiological conditions 29. But another writer METTL16 rapidly induces the splicing of the intron of *MAT2A*, encoding a SAM synthetase, and maintains low levels of intracellular SAM 30.

### m6A in mRNA nucleation

After alternative spicing, mature mRNA enters the cytoplasm from the nucleus for translation. m6A modification is also involved in this process; in essence, this kind of regulation utilizes the formation of steric resistance to ultimately target translation. m6A reader YTHDC1 is involved in the process of mRNA nuclear export. The methylated mRNA is recognized by the nuclear protein YTHDC1 and delivered to the nuclear mRNA export receptor NXF1 via interactions with the splicing factor and nuclear export adaptor protein SRSF3 31.

### m6A in mRNA translation

m6A modification can improve the translation efficiency through the binding of reader proteins to protein factors required in the translation process, and m6A modifications located in different RNA regions exert effects by various modes of action. METTL3 promotes translation by identifying 5′ UTR m6A and 3′ UTR m6A 32. Another model shows that METTL3 binds to eIF3, which interacts with mRNA cap-associated proteins, resulting in the formation of an mRNA loop. However, direct METTL3 tethering can promote translation only when bound to the 3′ UTR at a position near the stop codon 33. YTHDF1/2/3 are all reported to enhance translation, but the mechanism of YTHDF2 is still not clear 34-36. Via interactions with the translation elongation factor eEF2, YTHDF1 mediates the CDS m6A-enhanced translation elongation of Snail mRNA, although a previous study indicated that it also binds to *eIF3* in the 3′ UTR 34. YTHDF3 significantly promotes the binding of eIF3a to m6A residues within the 5′ UTR of *YTHDF3* mRNA to enhance cap-independent translation in breast cancer brain metastases 36.

### m6A and mRNA stability

m6A protein machinery is also essential for maintaining the stability of mRNA. Through different molecular mechanisms, m6A-containing transcripts can mediate RNA decay, which is induced primarily by m6A readers. YTHDF2-bound m6A mRNAs are degraded by at least two pathways. First, when a heat-responsive protein (HRSP)12-binding site and an RNase P/MRP (endoribonucleases)-directed cleavage site exist upstream and downstream of the YTHDF2-binding site, respectively, HRSP12 functions as an adaptor to bridge YTHDF2 and RNase P or MRP, eliciting the rapid degradation of YTHDF2- bound RNAs by an endoribonucleolytic cleavage pathway^37^. Second, via exosomes (3′-to-5′ exoribonuclease complex) and P bodies where the decapping complex and 5′-to-3′ exoribonuclease (XRN1) are enriched, YTHDF2 directly recruits the CCR4/NOT deadenylase complex to trigger deadenylation and subsequently initiates the degradation of m6A-containing mRNA 38-40. Interestingly, IGF2BPs get the opposite, they can maintain the stability of mRNA. IGF2BPs stabilize mRNAs by binding to RNA stabilizers, such as HuR, matrin 3 (MATR3), and poly(A)-binding protein cytoplasmic 1 (PABPC1) 15.

Currently, studies on the regulatory mechanism of m6A protein machinery are not complete, and subsequent studies need to study the specific mechanisms and find more RNA-binding proteins.

## Oogenesis and female infertility

Beginning during fetal life, mammalian oogenesis is completed after puberty 41. In the embryonic ovary, the oogonia change abruptly from successive mitotic divisions into meiosis and become arrested at MPI (meiotic prophase I) stage. Enclosed by pregranulosa cells, the early oocytes form the primordial follicles. After puberty, the oocytes resume meiosis to finish the first meiotic division. The follicles keep growing in size and putting on extra continuous layers of granulosa cells around them, but only the dominant follicle is chosen to produce the mature egg for ovulation. Then the eggs become arrested in meiotic metaphase II (MII) until fertilization 42. In the last few years, studies have shown that the m6A modifications are essential for oogenesis. The proof comes from that there was significant enrichment of differentially expressed m6A methylated genes in several signaling pathways associated with steroidogenesis, granulosa cell proliferation and follicular development 43. Recent studies have confirmed that m6A protein machineries are also involved in ovulation, including METTL3, METTL14, YTHDC1, YTHDC 2, YTHDF1, YTHDF2, YTHDF3 and KIAA1429 (**Table 1**).

The loss of METTL3 leads to failed mature gametes and impaired fertility, possibly as a result of m6A downregulation and interrupted expression of genes important for sex hormone synthesis and gonadotropin signaling pathway (e.g. *npr, igf3, star, 3βhsd, and cyp19a1a*) 44. Furthermore, the sex steroids 11-ketone testosterone and estradiol have significant regulatory effects on germ cells to promote gametogenesis and gamete maturation 45. The mRNA levels of METTL14 in L-ascorbic acid treated porcine oocytes were significantly reduced, which enhanced the ability of meiosis maturation and development of porcine oocytes 46. This result suggests that METTL14 may also play an important role in ovulation. The newly discovered KIAA1429 is a member of the family of m6A writers. KIAA1429-deficient germinal vesicle oocytes displayed abnormal apoptosis and proliferation of granulosa cells, as well as abnormal chromatin configuration and RNA metabolism 47. According to the above evidence, m6A writers plays an important role in oogenesis, but whether they can be used as a target for treating abnormal ovulation remains to be further studied.

Various studies have shown that ovulation cannot occur without YTH-domain including YTHDC1/2 and YTHDF1/2/3.^48^-^52^. The knockout of YTHDC1 leads to extensive selective polyadenylation in oocytes, changes the length of 3' Untranslated Region (3'-UTR), and eventually causes a lot of alternative splicing deficiency in oocytes, which hinders the development of oocytes and leads to the lack of secondary follicles or antral follicles in ovaries 48.

Interestingly, a recent study confirms that the adult female mice with YTHDC2 gene knockout were infertile due to the lack of developing follicles, and the fetal female germ cells could not carry out normal early pregnancy 50. m6A may regulate female germline stem cells self-renewal through m6A binding protein YTHDF1 51*.* The lack of YTHDF2 leads to the failure of m6A modified mRNA degradation, which affects the quality of oocytes 27*.* Moreover, double mutation of YTHDF2 and YTHDF3 resulted in impaired female gonad development 52, consistent with previous works proposing m6A and its protein machineries as regulators of gametogenesis 49. At present, the specific mechanism of the YTH family in ovulation is still unclear, and more studies are needed to find the specific genes regulated by the YTH-domain.

More interestingly, up-regulation of m6A is a high-risk factor of premature ovarian insufficiency (POI). Concretely, in patients with POI and mice model, the levels of m6A modified mRNA was significantly higher than that in the control group, while the expression of FTO was the opposite. However, the specific mechanism of m6A in POI still remains unclear up to now 53.

The role of m6A in ovulation has only been preliminarily explained, and there is still a lot of gaps, for example, the exact role of how m6A modification influences oogenesis at different developmental stages remains largely unknown, especially in humans, owing to inaccessibility of the early human germ line in vivo.

## m6A modification in female reproductive system neoplasms

**Figure 2:**
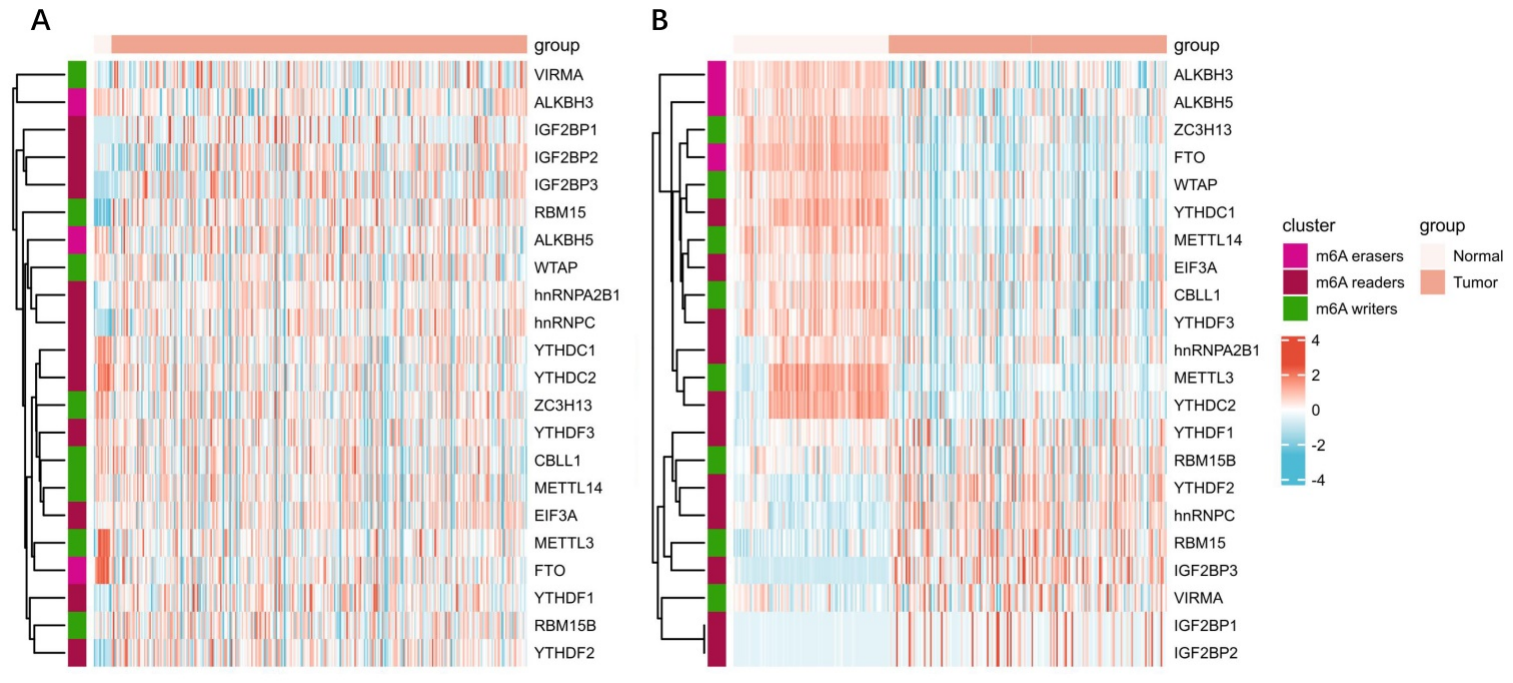
** Expression heatmap of m6A released genes in A) cervical cancer, and B) endometrial cancer**. On behalf of the heatmap of m6A related genes in cancers. The left part of the figure represents normal tissue, and right represents cancer tissue. The color of the grid in the heatmap represents the relative expression of the gene. All tumor tissue data were obtained from TCGA database and that for normal tissue came from the GTEx database. All the above analysis methods and R package were implemented by R version 4.0.3 and software packages ggplot2 and pheatmap.

**Figure 3:**
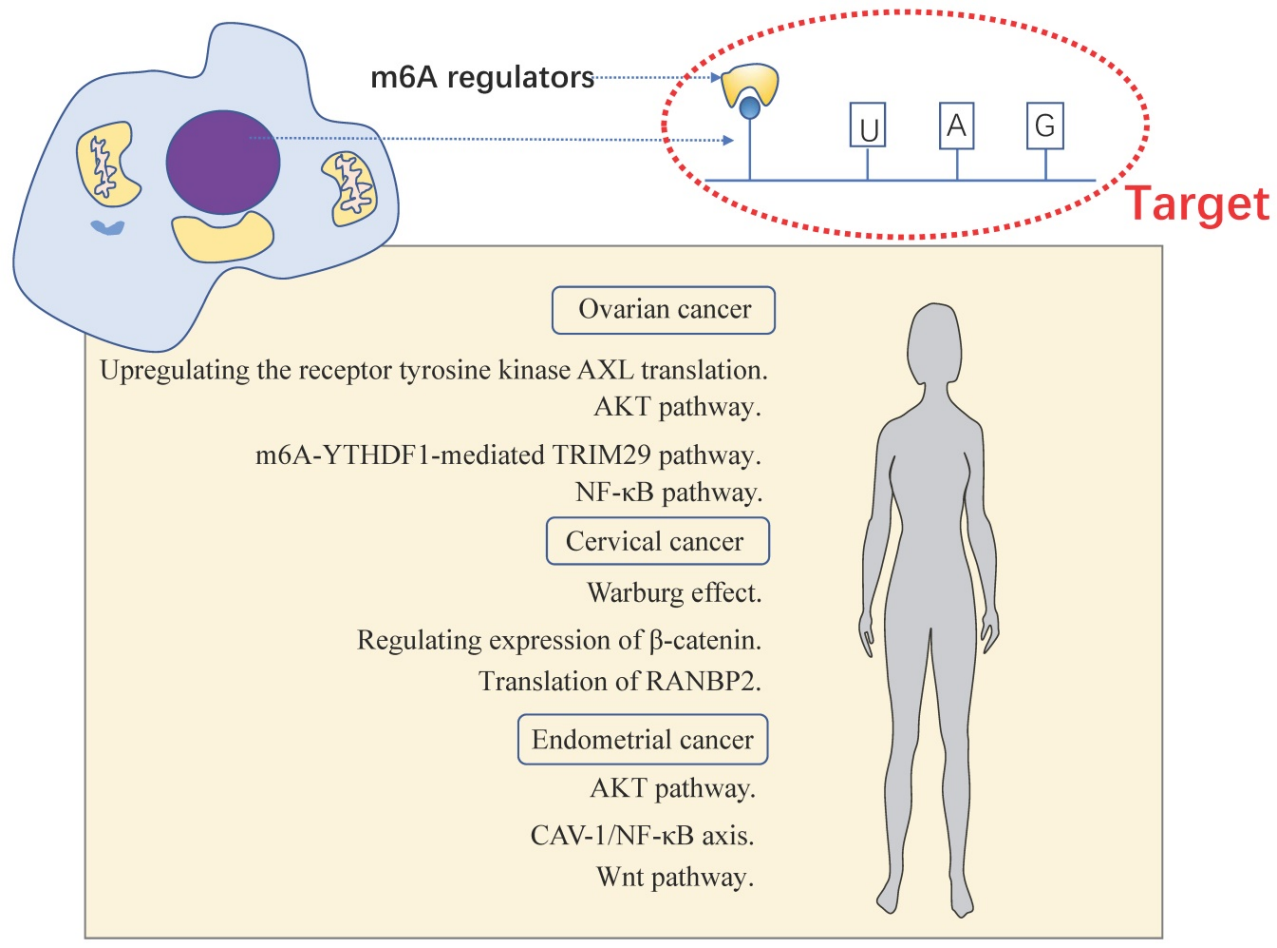
** The momentous biological pathways of m6A exerted in female reproductive system tumors.** Female reproductive system tumors including OC, CC and EC.

m6A has been shown to play an important role in many physiological processes and various cancers. Epithelial transcription of tumor cells promotes carcinogenesis by up-regulating or down-regulating the expressions of m6A “writer”, “reader”, and “eraser”. Same is true in female reproductive system neoplasms, including OC, CC and EC (**Table 2**). We analyzed the expression of the m6A protein machineries in cervical cancer and endometrial cancer using databases such as The Cancer Genome Atlas (TCGA) dataset and Genotype Tissue Expression (GTEx) dataset. Compared to normal tissue, bioinformatics analysis of multiple m6A protein machineries in cervical and endometrial cancer revealed that the expression of multiple m6A protein machineries varied in cancer tissues (**Figure 2A-B**). We found that the expression of m6A writers including METTL3 and METTL14 were down-expressed in EC, the same as reported in the literature. In CC, YTHDF1/2 were overexpressed. To some extent, this validates and complements the changes in m6A protein machineries reviewed in our literature. More importantly, the mechanism of m6A modification exerted in these tumors are also reviewed (**Figure 3**).

### Ovarian cancer

OC is the leading cause of death in women diagnosed with gynecological cancers. In general, it is also the fifth most frequent cause of death in women 54. Most OC patients are diagnosed at an advanced stage, so the selection or invention of an efficient diagnosis and screening method has become an effective measure for early detection of OC patients. The standard line of care treatment includes surgery and platinum-based chemotherapy. Moreover, current treatments for OC are associated with high recurrence rates and poor prognosis in some patients. Discovering efficient and safe diagnosis and treatment of OC has become a valuable research topic. In recent years, immune-checkpoint inhibitors (ICIs) have emerged in cancer therapy, but they do not seem to be ideal in OC. This may be related to the inhibitory effect of tumor microenvironment (TME) in the treatment of OC. The absence of an immune response in OC may reflect the inefficiency or absence of antigen presentation and adaptive immune response initiation. However, recent research into the tumor microenvironment seems to provide insight for a breakthrough on that. Recent study shows METTL3 plays an important role in TME. METTL3 depletion in macrophages reshaped the TME by increasing M1- and M2-like tumor-associated macrophages (TAMs) and regulatory T (Treg) cell infiltration in vivo, resulting in tumor growth, metastasis, and drug resistance. Mechanistically, knockout of METTL3 in macrophages inhibits the YTHDF1-mediated SPRED2 translation to upregulate ERK expression to activate NF-κB and STAT3 signaling 55. Whether this effect also exists in ovarian cancer, as well as other neoplasm of reproductive system, is worth further exploration. Also, Luo *et al*.^56^ found m6A affected the process of antigen presentation in the immune system and played an important role in TME cell infiltration in OC.

Some m6A protein machineries and methylated gene loci have been found in OC, which may be used as therapeutic targets or prognostic markers in the future. However, m6A protein machinery is worrying as a treatment target because an m6A protein machinery can regulate the metabolic process of multiple gene transcription products, which leads to its lower specificity and more adverse reactions. Further study can be focused on exploring the specific role of m6A modification in TME. And then the treatment of OC may be promoted to a new height.

METTL3 was frequently upregulated in OC and that a high level of METTL3 was significantly associated with higher tumor grade. Hua *et al*. 28 found that stable overexpression of METTL3 *in vitro* significantly increased cellular proliferation, focus formation, motility, invasion, and tumor formation in nude mice. However, silencing METTL3 expression in cell lines with short hairpin RNA effectively inhibited its oncogenic function. Mechanism analysis shows that METTL3 promotes ovarian carcinoma growth and invasion through upregulating the receptor tyrosine kinase AXL translation and epithelial to mesenchymal transition 28. In METTL3 knockdown OC cells, apoptosis rates increased, which may have been mediated by activating the mitochondrial apoptosis pathway. METTL3 knockdown downregulated the phosphorylation levels of AKT and the expression of the downstream effector Cyclin D1. These results suggested that METTL3 may serve an oncogenic function in the progression of human OC cells partially through the AKT signaling pathway 57. METTL3 knockdown reduced m6A enrichment of the genes associated with OC including EIF3C, AXL, CSF-1, FZD10 *in vitro*. And the high expressed METTL3 indicated poor malignancy and survival of endometrioid epithelial OC via modulating the aberrant m6A RNA methylation 58.

YTH-domain affects the development of OC directly or indirectly through m6A modification. YTHDF1 can promote OC by enhancing the expression of m6A modified mRNA of some specific genes. And genes identified include TRIM29 and EIF3C 59, 60.

Li *et al*. 61 demonstrated that YTHDF2 promoted proliferation and migration, inhibited apoptosis, and reduced global mRNA m6A levels of epithelial OC (EOC) cell lines. YTHDF2 has been identified as a novel substrate for the enzyme FBW7 which is markedly down-regulated in OC tissues and is negatively correlated with the prognosis. FBW7 counteracts the tumor-promoting effect of YTHDF2 by inducing proteasomal degradation of the latter in OC 62. Additionally, EOC can be negatively regulated by miR-145, resulting in cell proliferation inhibition 61. Similarly, the expression of YTHDF3 was positively correlated with OC malignancy, but this was only based on the validation of bioinformatics 63.

FTO inhibited the self-renewal of ovarian cancer stem cell (CSC) and suppressed tumorigenesis *in vivo*. Integrative RNA-sequencing and m6A mapping analysis revealed significant transcriptomic changes associated with FTO overexpression and m6A loss involving stem cell signaling, RNA transcription, and mRNA splicing pathways. By reducing m6A levels at the 3'-UTR and the mRNA stability of two phosphodiesterase genes (PDE1C and PDE4B), FTO augmented second messenger 3',5'-cyclic adenosine monophosphate signaling and suppressed stemness features of OC cells 64. However, ALKBH5 got the opposite result. NANOG, one dispensable gene in cell proliferation, whose expression was up-regulated by ALKBH5, was involved in the tumorigenesis of OC 65.

Bioinformatics indicate that m6A protein machineries are associated with the prognosis of OC patients 66. Regression models identified that prognosis is associated with HNRNPA2B1, KIAA1429, and WTAP 67. However, m6A protein machineries are still not used to determine the prognosis of OC patients clinically, maybe the accuracy still needs to be improved.

From above all, METTL3, YTHDF1/2/3, ALKBH5 play positive role in the occurrence and development of OC, while FTO is a tumor suppressor. (**Table 2**) More studies of m6A modification in OC other reproductive tumors lay a foundation for us to have a clearer understanding of the pathogenesis of OC, which may be conducive to better prevention and treatment. But it is also important to know that there are many categories of OC including epithelial carcinoma of the ovary, malignant germ cell tumor of ovary and malignant sex cord-stromal tumors. It still needs to be considered whether the effect of m6A modification is consistent across different categories of OC. As the most malignant tumor in OC, whether m6A modification is participated in hyaline cell carcinoma of ovary (one kind of epithelial carcinoma of the ovary) is still not clear. All of these can be contained in the future study.

### Cervical cancer

Cervical cancer (CC) is the most common gynecological tumor worldwide. Persistent infection of high-risk HPV-induced chronic inflammation is considered to be an important risk factor for CC. TME also plays an important role in the progress of the tumorigenesis, development, and prognosis of CC 68. CC has higher tumor mutation burden (TMB) level and inflammatory gene expression, suggesting that there may be a continuous functional suppression of the immune response, better response to ICIs. On this basis, they respond better to PD-1/PD-L1 or CTLA-4 inhibitors. However, the treatment will be less effective because of immune avoidance or immunosuppressive signaling pathways. These mechanisms include adaptive immune response, loss of tumor antigen expression, insensitivity to antibiotics, and imbalance of metabolites and cytokines 69, which may affect the therapeutic effectiveness of ICIs. Recent study shows m6A modification participates in the expression of PD-L1 indirectly. METTL14 can induce the expression of seven in absentia homolog 2 (Siah2), which has been involved in tumorigenesis and cancer progression. Siah2 knockdown inhibited T cells expansion and cytotoxicity by sustaining tumor cell PD-L1 expression. Analysis of specimens from patients receiving anti-PD1 immunotherapy suggested that tumors with low Siah2 levels were more sensitive to anti-PD1 immunotherapy 70. Whether this is effective in CC still remains to be studied.

METTL3 can promote the proliferation and invasion of CC cells 71. So far, several studies have explored the specific functions of METTL3 in CC. Wang *et al*. 72 found METTL3 was significantly upregulated in CC tissue and cells, which was closely correlated with the lymph node metastasis and poor prognosis of CC patients. Mechanistically, METTL3 targeted the 3'-UTR of hexokinase 2 (HK2) mRNA, again recruited YTHDF1 to enhance HK2 stability, promoting Warburg effect of CC 72. Interestingly, the m6A modification of pyruvate dehydrogenase kinase 4 is mediated by METTL3.Via binding with YTHDF1 and IGF2BP3, the translation of m6A modified PDK4 mRNA is enhanced, promoting the glycolysis of cancer cells, resulting in the growth and progression of CC 73. A recent study reveals a different mechanism, METTL3 can promote CC aggressiveness by repressing the activity of miR-193b, which can regulate the expression of “CCND1” positively 74.

FTO was frequently overexpressed in human CC tissues and highly correlated with CC progression. FTO serves as an oncogenic regulator for CC cells proliferation and migration. Mechanistically, FTO directly interacted with E2F1 and Myc mRNAs and inhibition FTO dramatically impaired these two important oncogenes translation, thus suppressed CC cells proliferation and migration 75. However, different researchers hold different opinions on the relationship between FTO and CC. Zhou *et al*. 76 discovered FTO enhances the chemo-radiotherapy resistance both in vitro and in vivo through regulating expression of β-catenin by reducing m6A levels in its mRNA transcripts.

In CC, YTHDF1 was overexpressed, and it was closely associated with poor prognosis 77. YTHDF1 regulated RANBP2 translation in an m6A-dependent manner, which potentiated the growth, migration and invasion of CC cells.

Currently, m6A protein machineries found in cervical cancer including METTL3, YTHDF1 and FTO all play a role in promoting the occurrence of cancer (**Table 2**), and whether other machineries are involved in CC can be explored in the future.

### Endometrial cancer

Endometrial carcinoma (EC) is the most frequent gynecological malignancy in developed countries and requires a relatively invasive diagnostic evaluation and operative therapy as the primary therapeutic approach 78. It has been confirmed that significant changes in the endometrial cancer immune microenvironment, such as the number of CD8^+^ T cells decreases 79. In addition, Garzetti *et al.* 80 suggested that locally advanced stage I and II ECs had significantly lower mean values of NK cell activity compared with healthy controls, which means that NK cells are less able to kill tumor cells. Figuring out how to enhance the immune response to EC by regulating tumor immunosuppressive microenvironment has become the focus of future EC immunotherapy research. Dong *et al*. 81 found that macrophage-specific knockout of an m6A methyltransferase METTL14 drives CD8^+^ T cell differentiation along a dysfunctional trajectory, impairing CD8^+^ T cells to eliminate tumors, which was found in colorectal cancer. But it also provides insights into EC. It shows that m6A modification may participate in tumor immunosuppressive microenvironment of EC. Future studies could also explore whether m6A modifications play a role in the EC-induced immunosuppressive microenvironment, so as to provide more possibilities for immunotherapy. Up to now, research on M6A modifications and EC relationships has made preliminary progress, but there are many phenomena still difficult to explain.

The downregulation of METTL3 enhance the proliferation and tumorigenesis of EC through AKT pathway. That resulted in changes in the expression levels of PHLPP2 and mTORC2. PHLPP2, a phosphatase regulating AKT phosphorylation, and mTORC2, a kinase that phosphorylates AKT 82. Reductions in m6A methylation lead to decreased expression of the negative AKT regulator PHLPP2 and increased expression of the positive AKT regulator mTORC2 and the activity of AKT pathway, promoting abnormal cell proliferation. 82.WTAP, one of the most important enzymes catalyzing generation of m6 A on mRNA could methylate 3'-UTR of CAV-1 and downregulate CAV-1 expression to activate NF-κB signaling pathway in EC, which promoted EC progression 83.

The expression of KIAA1429 observed in EC was significantly decreased, leading to the reduction of m6A levels 84. KIAA1429 gene expression is associated with cellular nucleic metabolism. It was discovered that KIAA1429 contributed to liver cancer progression through N6-methyladenosine-dependent post-transcriptional modification of GATA binding protein 3, which is a highly conserved, essential transcription factor expressed in a number of tissues 85. But in EC the mechanism is still not explicit.

m6A “erasers” including FTO and ALKBH5 can promote EC through enhancing the mRNA stability and protein expression of some important genes. FTO can decrease HOXB13 mRNA decay and increase HOXB13 protein expression, promoting Wnt signaling pathway activation and the expression of downstream proteins, leading to tumor metastasis and invasion 86. ALKBH5 demethylated target transcripts IGF1R and enhanced IGF1R mRNA stability, consequently promoting IGF1R translation and activating IGF1R signaling pathway, eventually enhancing proliferation and invasion of EC 87.

YTHDC1 knockdown promoted the proliferation and invasion of EC cells 88. But YTHDF2 was identified to inhibit the proliferation and invasion of EC cell lines. Mechanistically, the m6A reader YTHDF2 bind the methylation sites of target transcripts Insulin Receptor Substrate 1 (IRS1) and promoted IRS1 mRNA degradation, consequently inhibiting the expression of IRS1 and inhibiting IRS1/AKT signaling pathway, finally inhibit the tumorigenicity of EC 89. IRS1 plays a key role in cancer cell proliferation and mediates the resistance to anticancer drugs 90. IGF2BP1 expression increased in EC, and high expression of this protein correlated with poor prognosis 91. IGF2BP1 overexpression can promote cell proliferation and regulate the tumor cell cycle and cancer progression, both in vivo and in vitro. Mechanistically, IGF2BP1 can recognize m6A sites in the 3'-UTR of Paternally Expressed Gene 10 (PEG10) mRNA and recruits polyadenylate-binding protein 1 (PABPC1) to enhance PEG10 mRNA stability, which consequently promotes PEG10 protein expression. Additionally, it would appear that a large number of PEG10 proteins bind p16 and p18 gene promoter sequences, thereby repressing expression and accelerating the cell cycle 92. Another study showed that IGF2BP1 was enriched in microRNAs in cancer pathway, contributing to the progression of EC 93.

METTL3, IGF2BP1, WTAP, FTO, ALKBH5 are involved in the occurrence and development of EC. However, as a tumor suppressor in EC, YTHDF2 can inhibit the tumorigenicity of EC (**Table 2**).

## Other female reproductive system diseases and m6A

m6A modification is not well studied in other female reproductive system diseases including adenomyosis, endometriosis, polycystic ovary syndrome and premature ovarian failure. The future research should focus on further exploring whether m6A modification plays roles in the occurrence and development of these diseases. And then the researchers can further explore the specific target of m6A modification.

### Adenomyosis

As a common uterine disease, adenomyosis is characterized by abnormal findings of endometrial epithelial cells and stromal fibroblasts in the myometrium, where they cause proliferation and hypertrophy of surrounding smooth muscle cells^94^. At present, the pathological mechanism of adenomyosis is not very clear, which makes it difficult to find a good diagnosis and treatment. Zhai *et al*. 95 found that m6A protein machineries contributed to the pathogenesis of adenomyosis. Bioinformatics analysis showed that METTL3, ZC3H13, FTO, and YTHDC1 were significantly reduced in patients with adenomyosis, which caused decreased m6A levels. Possible target genes are cadherin 3(CDH3), sodium channelβ-subunit 4 (SCN4B), and placenta-specific protein 8 (PLAC8), which are involved in cell adhesion, muscle contraction and immune response in the myometrium of adenomyosis patients were also validated 95. Their findings undoubtedly provide new ideas for the diagnosis and treatment of adenomyosis, but it is worth noting that the above experiments have not been verified in animal models, and more research is still needed to find effective drug therapeutic targets.

### Endometriosis

Endometriosis patients have lower levels of m6A in the endometrium than normal endometrium, and this reduction is due to lower levels of METTL3. Li *et al*. 96 found that METTL3 knockdown promotes migration and invasion of human endometrial stromal cells (HESCs), while METTL3 overexpression has the opposite effect, suggesting that METTL3 knockdown may promote the development of endometriosis by promoting cell migration and invasion. In addition, they found the pathway that mediated this reaction. Specifically, suppressive METTL3 enhances cell migration and invasion by attenuating DGCR8-mediated maturation of pri-miR126 in an m6A-dependent manner, thus contributing to endometriosis development. Bioinformatics analysis also supports this finding, METTL3, YTHDF2, YTHDF3, HNRNPA2B1, HNRNPC and FTO are found decreased in ectopic endometrium. HNRNPA2B1 and HNRNPC may be associated with immune response and can be used as useful biomarkers in the diagnosis of endometriosis 97. Currently, there are few studies on the role of m6A protein machineries in endometriosis, but existing studies have shown that they play a significant role in endometriosis, and some new drugs for the treatment of endometriosis can be developed by targeting m6A protein machineries.

### Polycystic ovary syndrome

The pathophysiological feature of polycystic ovary syndrome (PCOS) is granulosa cells (GCs) dysfunction. A recent study found that m6A levels were elevated in luteinized granulosa cells in PCOS patients. It is found that FOXO3 mRNA lacked m6A modification in luteinized granulosa cells from PCOS patients. Selective knockout of m6A methyltransferase or demethylase altered FOXO3 expression in luteinized GCs in the control group, but not in PCOS patients. These results suggest that m6A-mediated FOXO3 transcription is absent in luteinized GCs in PCOS patients. Forkhead Box O3 (FOXO3) plays important roles in diverse cellular processes including apoptosis, metabolism, cell proliferation and cell survival^98^. This study sheds light on the potential mechanism of PCOS.

### Premature Ovarian Failure

As one of the most commonly used alkylated anticancer drugs, Cyclophosphamide (CTX) is associated with premature ovarian failure. Huang *et al*. 99 found that CTX may affect ovarian function by affecting m6A levels. They found CTX increased m6A levels in a time and concentration- dependent manner. Except for RBM15 and WTAP, the expression level of RNA methyltransferase in CTX treatment group was significantly higher than that in control group in a time-dependent and concentration-dependent manner. CTX significantly inhibited the expression of RNA demethylase FTO in a time-dependent and concentration-dependent manner, but did not significantly inhibit ALKBH5 99. Although they performed in vivo and in vitro studies, they did not identify the specific mechanism by which CTX affects m6A protein machineries or the specific target of premature ovarian failure.

## Perspective

Despite the researches of m6A in reproduction have make dramatic roles in recent years, a large number of challenges still exist. Firstly, most of the conclusions are derived from bioinformatics analysis or in vitro experiments, in vivo experiments are rarely involved. Secondly, it is possible some enzymes that modify m6A have not been identified. Thirdly, the mechanisms of m6A protein machineries in some infertility diseases are still unclear, more efforts are needed to explore the specific mechanism of m6A in the various pathways that regulate gene expression. Fourthly, the specific sites of m6A modification are rarely reported, which greatly limits clinical transformation. Fifthly, studies showed that regulation of m6A level and its protein machineries may be potential therapeutic targets for some reproduction diseases, but lack of the specific applications in clinical practice with a large sample size, and the safety, effectiveness, even the corresponding side effects are largely unknown. Sixthly, it is uncertain whether m6A modification plays the same role in different tumor subtypes. All of these issues should be addressed.

## Conclusions

RNA modification, especially m6A modification, has become a hot topic in recent years. m6A is extremely important for mRNA metabolism at different stage, from processing in the nucleus to translation and decay in the cytoplasm. In this review, we summarized that m6A modification and its regulators played a key role in the occurrence and development of oogenesis and female reproductive system diseases. The change of m6A protein machineries contributed to the proliferation and aggressiveness of tumors. With the introduction of m6A detection technology into large-scale commercial use, the m6A level and its protein machineries have become more possible for the diagnosis of oogenesis and female reproductive system diseases. In addition, we reviewed the regulatory mechanisms and target genes that have been discovered so far, providing prospects for the study of related drugs and treatments.

## Figures and Tables

**Table 1:** Roles of m6A protein machineries and biological mechanisms exerted in oogenesis.

Type	Regulator	Role	Mechanism	Reference
Writers	METTL3	METTL3 loss caused failed mature gametes and impaired fertility	Interrupted expression of genes important for sex hormone synthesis and gonadotropin signaling pathway	45
	METTL14	Reduced METTL14 enhanced the ability of meiosis maturation and development of porcine oocytes.	/	46
	KIAA1429	KIAA1429-deficient germinal vesicle oocytes displayed abnormal apoptosis and proliferation of granulosa cells	The alternative splicing of genes associated with oogenesis is affected.	47
Readers	YTHDC1	YTHDC1 deficient oocytes are impeded at the primary follicular stage.	A large number of alternative splicing deficiency in oocytes	48
	YTHDC2	Adult female mice with YTHDC2 gene knockout were infertile	YTHDC2 suppressed expression of the meiotic markers and affected the percent of FGCs at zygotene	50
	YTHDF2/3	Double mutation of YTHDF2 and YTHDF3 resulted in impaired female gonad development	Failure of m6A modified mRNA degradation	52
Erasers	FTO	The decrease of FTO mRNA and protein expression caused high risk POI	/	53

FGCs: female germ cells; POI: premature ovarian insufficiency;

**Table 2:** Roles of m6A protein machineries and biological mechanisms exerted in female reproductive system tumor.

Cancers	Regulator	Role in cancer	Mechanism	Functional classification	Reference
Ovarian cancer	METTL3	Oncogene	Through upregulating the receptor tyrosine kinase AXL translation and epithelial to mesenchymal transition.	Promoting OC growth and invasion	28
	METTL3	Oncogene	Through AKT pathway	Functioning in the progression of human OC cells	57
	METTL3	Oncogene	Via modulating the aberrant m6A RNA methylation on genes including EIF3C, AXL, CSF-1	Indicating poor malignancy and survival of endometrioid epithelial OC	58
	YTHDF1	Oncogene	Through m6A-YTHDF1-mediated TRIM29 pathway	Indicating a poor prognosis in the cisplatin-resistant OC cells	59
	YTHDF1	Oncogene	Enhancing EIF3C translation by binding to m6A-modified EIF3C mRNA	Indicating poor prognosis	60
	YTHDF2	Oncogene	FBW7 can suppress OC development by targeting YTHDF2	Promoting proliferation and migration of OC	62
	YTHDF2	Oncogene	miR-145 can repress the proliferation and migration of OC by suppress YTHDF2	Promoting proliferation and migration of OC	61
	YTHDF3	Oncogene	/	Increasing the pathological grade of OC	63
	FTO	Tumor Suppressor	By blocking cAMP signaling	FTO inhibited the self-renewal of ovarian CSC and suppressed tumorigenesis in vivo	64
	ALKBH5	Oncogene	Through NF-κB pathway.	Participating in the tumorigenesis of OC	65
Cervical cancer	METTL3	Oncogene	Through enhancing Warburg effect	Promoting the proliferation and invasion of CC cells	72
	METTL3	Oncogene	Through enhancing the m6A modification of PDK4	Resulting in the growth progression of CC	73
	METTL3	Oncogene	By repressing the activity of miR-193b, which can regulate the expression of CCND1 positively	Promoting CC aggressiveness	74
	FTO	Oncogene	Through interacting with E2F1 and Myc mRNAs	Promoting CC cells proliferation and migration.	75
	FTO	Oncogene	Through regulating expression of β-catenin	Enhancing the chemo-radiotherapy resistance both in vitro and in vivo	76
	YTHDF1	Oncogene	Through regulating RANBP2 translation	Indicating poor prognosis	77
Endometrial cancer	METTL3	Oncogene	Through AKT pathway	Promoting the proliferation and tumorigenicity of EC	82
	IGF2BP1	Oncogene	Stabilizing PEG10 mRNA in an m6A-dependent manner	Indicating poor prognosis	92
	WTAP	Oncogene	Via CAV-1/NF-κB axis	Promoting EC progression.	83
	FTO	Oncogene	Through activating Wnt signaling pathway	Promoting EC metastasis	86
	ALKBH5	Oncogene	Through enhancing IGF1R mRNA stability and promoting IGF1R translation	Promoting the proliferation and tumorigenicity of EC	87
	YTHDF2	Tumor Suppressor	Via downregulating the expression of IRS1 methylated with m6A	Inhibiting the tumorigenicity of EC	89

CSC: cancer stem cell; OC: ovarian cancer; CC: cervical cancer; EC: endometrial cancer; IRS1: Insulin Receptor Substrate 1; PDK4: pyruvate dehydrogenase kinase 4; PEG10: paternally expressed gene 10

## Abbreviations

OC: ovarian cancer
CC: cervical cancer
EC: endometrial cancer
WTAP: Wilms-tumor associating protein
VIRMA/ KIAA1429: Vir-like m6A methyltransferase-associated
FTO: Obesity-associated protein
ALKBH5: alkB homolog 5
POI: premature ovarian insufficiency
CSC: cancer stem cell
EOC: epithelial OC
HK2: hexokinase 2
IRS1: Insulin Receptor Substrate 1
PEG10: Paternally Expressed Gene 10
PABPC1: polyadenylate-binding protein 1
AF: angiogenic factor
